# TLR4 Activation Promotes Bone Marrow MSC Proliferation and Osteogenic Differentiation via Wnt3a and Wnt5a Signaling

**DOI:** 10.1371/journal.pone.0149876

**Published:** 2016-03-01

**Authors:** Xiaoqing He, Hai Wang, Tao Jin, Yongqing Xu, Liangbin Mei, Jun Yang

**Affiliations:** 1 The Third Military Medical University, Chongqing, China; 2 Department of Orthopaedic Surgery, Kunming General Hospital of Chengdu Military Command, Kunming, China; University of Udine, ITALY

## Abstract

Mesenchymal stem cells (MSCs) from adult bone marrow maintain their self-renewal ability and the ability to differentiate into osteoblast. Thus, adult bone marrow MSCs play a key role in the regeneration of bone tissue. Previous studies indicated that TLR4 is expressed in MSCs and is critical in regulating the fate decision of MSCs. However, the exact functional role and underlying mechanisms of how TLR4 regulate bone marrow MSC proliferation and differentiation are unclear. Here, we found that activated TLR4 by its ligand LPS promoted the proliferation and osteogenic differentiation of MSCs *in vitro*. TLR4 activation by LPS also increased cytokine IL-6 and IL-1β production in MSCs. In addition, LPS treatment has no effect on inducing cell death of MSCs. Deletion of TLR4 expression in MSCs completely eliminated the effects of LPS on MSC proliferation, osteogenic differentiation and cytokine production. We also found that the mRNA and protein expression of Wnt3a and Wnt5a, two important factors in regulating MSC fate decision, was upregulated in a TLR4-dependent manner. Silencing Wnt3a with specific siRNA remarkably inhibited TLR4-induced MSC proliferation, while Wnt5a specific siRNA treatment significantly antagonized TLR4-induced MSC osteogenic differentiation. These results together suggested that TLR4 regulates bone marrow MSC proliferation and osteogenic differentiation through Wnt3a and Wnt5a signaling. These finding provide new data to understand the role and the molecular mechanisms of TLR4 in regulating bone marrow MSC functions. These data also provide new insight in developing new therapy in bone regeneration using MSCs by modulating TLR4 and Wnt signaling activity.

## Introduction

Adult bone marrow mesenchymal stem/stromal cells (MSCs) are multipotential stem cells, and have the ability to differentiate into osteoblasts, chondrocytes, tenocytes, neurons, adipocytes, and skeletal myocytes [1,2]. Due to the multipotent of MSCs, they have become a therapeutic option for several pathologies including osteogenesis imperfecta, myocardial infarction, and wound healing. MSCs play an important role in bone remodeling because they can be induced to differentiate into osteoblasts. Published data have revealed that bone marrow derived MSCs can repair bone defects in animal models [3,4]. In addition to their multipotential plasticity, MSCs play a critical role in regulating immune responses in a manner that depends on their state of activation [5,6]. It is also an important cell to form the critical microenvironment for bone regeneration after injury [7]. Therefore, it is important to reveal the molecular mechanisms that regulate the MSC function including survival, proliferation, differentiation and cytokine secretion.

Toll-like receptor 4 (TLR4) are the best studied immune sensors of invading microbes. It is broadly distributed on cells throughout the immune system. Activation of TLR4 is essential for inducing the immune responses, and enhances adaptive immunity against pathogens [8,9]. It has been revealed that MSCs derived from adult bone marrow also express functional TLR4 [10,11,12,13]. Activation of TLR4 signaling in MSCs may influence their survival, differentiation, proliferation, migration and pro-inflammatory cytokine secretion [10,14,15]. TLR4 recognizes lipopolysaccharides (LPS) from gram-negative bacteria. It has been proved that LPS can protect MSCs from oxidative stress-induced apoptosis and enhance proliferation of MSCs via TLR4 and PI3K/Akt signaling [12]. LPS was also found to promote the osteogenic differentiation in adult human MSCs [14]. However, due to conflicting reports on various effects of TLR4 ligands on MSCs [12,13,14,16], further studies are still needed to explore the cellular biological changes of MSCs after TLR4 activation in different models. Importantly, the molecular mechanisms by which TLR4 regulating MSC proliferation and differentiation are largely unknown.

Wingless proteins (Wnt) are a family of cysteine-rich glycoproteins that regulate embryonic development, cell proliferation, migration, differentiation, and death [17,18]. Recently, Wnt signaling has been revealed to function in controlling the cell fate specification and differentiation of MSCs [19]. Previous researches have shown that Wnt signaling has the capacity to regulate the proliferation and migration of MSCs [20,21]. Moreover, Wnt signaling has also been found involved in the osteogenic and adipogenic differentiation process in human MSCs [22]. Till now, exist evidence have identified that Wnt3a, Wnt5a, Wnt6, Wnt10a, and Wnt10b signaling are all involved in controlling the stem cell properties of MSCs [20,23,24,25]. Wnt5a has been shown to be involved in the induction of MSC osteogenesis and suppression of adipogenesis [23]. Wnt3a promotes proliferation and suppresses osteogenic differentiation of adult human MSCs [20]. Wnt10b has been proposed to influence the decision of MSCs to give rise to either an adipocyte or an osteoblast. Knockdown of endogenous Wnt6 is associated with increasing preadipocyte differentiation and impaired osteoblastogenesis [25]. However, little is known about the interactions between TLR4 and Wnt signaling and their function in regulating MSC proliferation and differentiation.

In the present work, we used TLR4 ligand LPS to activate TLR4 in bone marrow MSCs. We studied the effects of TLR4 activation on the survival, proliferation, osteogenic differentiation, and cytokine production of bone marrow originated MSCs *in vitro*. We found that TLR4 activation promoted the proliferation and osteogenic differentiation of MSCs. In addition, we examined the involvement of Wnt signaling in this process, and found that Wnt3a and Wnt5a play critical role in TLR4-induced MSC proliferation and osteogenic differentiation. Our experiments provide new data to understanding the role and the molecular mechanisms of TLR4 in regulating bone marrow MSC functions.

## Methods

### Cell culture, identification, differentiation and LPS treatment

All the animal care and procedures were in accordance with the guidelines of the National Institutes of Health, and were approved by the Committee on Use of Live Animals for Research of the Third Military Medical University.

Bone marrow cells were obtained from femur and tibia of 6- to 8-week-old male C57BL/6J WT mice and TLR4^-/-^ mice (The Jakson lab., Strain: B6.B10ScN-Tlr4^lps-del^/JthJ). The cells were filtered through 40 μm nylon mesh, and centrifuged at 1000 rpm for 5 min. The buoyant adipocytes were removed by vacuum aspiration. The cell pellet was resuspended in Dulbecco’s modified Eagle’s medium (DMEM) supplemented with 10% fetal bovine serum (FBS), 2 mM glutamine, and 1% penicillin and streptomycin (Invitrogen, USA). The cells were cultured in 10 cm dishes and nonadherent cells were removed after 24 hours. Culture medium was changed every 3 days. Upon confluence, the cells were dissociated using 0.25% trypsin (Invitrogen, USA) and reseeded. For clone isolation, cultured cells were seeded in 96-well plates by limited dilution. Individual clones were picked and expanded for further experiments. Cells were used with in the 10th passage [26].

The MSC phenotype was defined using flow cytometric analysis with the following monoclonal antibodies: anti-CD31-PE (R&D, USA), anti-CD45-FITC (R&D, USA), anti-CD29-PE (Immunotech, France), and anti-CD44-PE (BD Biosciences, USA).

For osteogenic differentiation, cells were seeded into 24-well plates and allowed to attach overnight. The following day (day 0) growth medium was replaced with osteogenic differentiation medium, which is DMEM supplemented with 10% FBS, 100 nM dexamethasone (Sigma, USA), 10 mM β-glycerophosphate (Sigma, USA) and 100 mM vitamin C. Medium was replaced every 3 or 4 days[27].

Lipopolysaccharide (LPS, Sigma, USA) was added to the culture medium at a final concentration of 0, 10, 100, 1000 ng/ml, and treated for indicated times.

### Cell apoptosis measurement

MSCs were detached by trypsin treatment, washed, and then assessed for Annexin V (Invitrogen) and Propidium Iodide (PI) staining. Flow cytometric analysis was used to detect early apoptotic cells Annexin V-positive/PI-negative cells and late apoptopic or necrotic Annexin V-positive/PI-positive cells.

### Cell proliferation

The effect of LPS on the proliferation of MSCs was studied by a Colorimetric Cell-counting Kit (CCK-8, Dojindo, Japan) assay and EdU incorporation according to the manufacturer’s instruction. The EdU^+^ cells were counted in 10 different fields of each condition by an independent observer in a blinded manner using a 20 x objective. Three independent experiments were performed for every condition. The EdU^+^ were expressed as a percentage of total cells determined by Hoechst33342 staining [28].

### Alkaline phosphatase activity and alizarin red staining

ALP activity was detected using an ALP kit according to the manufacturer's instructions (Sigma-Aldrich). ALP activity was normalized to protein concentration of the cell lysate. For evaluation of mineralization, cells were induced for 7, 15, 20 to 25 days, fixed with 4% paraformaldehyde and stained with 2% Alizarin red (Sigma-Aldrich). For alizarin red quantification, the deposits were extracted with 10% acetic acid and 20% methanol solution. Light absorbance by the extracted dye was measured in 450 nm [29].

### Real-time PCR

Total RNA was isolated from cell pellets using the TRIzol reagent (Takara, Japan). cDNA was synthesized using a Sensiscript Revers Transcript Kit (Qiagen, Germany) according to the manufacturer's instructions. mRNA expression of the target genes was detected using the following primers: TLR4 fwd 5’- gcaggtggaattgtatcgcc -3’and rev 5’—tgctcaggattcgaggcttt—3’, Wnt3a fwd 5’—ggctcctctcggatacctct—3’ and rev 5’—gggcatgatctccacgtag—3’, Wnt5a fwd 5’—tggagaaggtgcgaagacag—3’ and rev 5’—cgtctctcggctgcctattt—3’, Wnt6 fwd 5’—ggtagtcctagagggccagg—3’ and rev 5’—cactccgtcatgccttggta—3’, Wnt10a fwd 5’—catcgggctgaagtgactct—3’ and rev 5’- ccggagcttctttttcccca -3’, Wnt10b fwd 5’—tcctccactacagcccagaa—3’ and rev 5’- cctcccaagagcctgacaag—3’, IL-1β fwd 5’—gcaactgttcctgaactcaact—3’ and rev 5’- atcttttggggtccgtcaact—3’, IL-6 fwd 5’—tagtccttcctaccccaatttcc—3’ and rev 5’—ttggtccttagccactccttc—3’, TNF-α fwd 5’—acggcatggatctcaaagac—3’ and rev 5’—agatagcaaatcggctgacg—3’, and GAPDH fwd 5’—atacggctacagcaacaggg- 3’ and rev 5’—gcctctcttgctcagtgtcc—3’. Real-time PCR was performed on a CFX96^TM^ real-time system using SYBR Master Mix (Takara, Japan). All gene expression values were normalized to the housekeeping gene GAPDH. The relative expression levels of the target genes were calculated using the control as a reference.

### Western blot analysis

Adherent cells were scraped from the culture dishes and total protein was extracted from the cell pellet with RIPA lysis buffer (Thermo Scientific, USA). Western blot analysis was performed and quantified with an Odyssey system (LI-COR, USA) as previously described [30]. The primary antibodies used for Western blot analysis were monoclonal mouse anti-TLR4 antibody (Santa Cruz Biotechnology, USA) and monoclonal mouse anti-GAPDH antibody (Abcam, USA) The secondary antibodies used were Odyssey-specific IRDye 680 or 800 donkey anti-mouse (1:5000; LI-COR, USA).

### Enzyme-linked Immunosorbent Assay (ELISA)

MSCs were seeded in 24-well culture plates. After the cells were treated with LPS for the indicated time or pretreated with specific siRNAs as indicated, the culture supernatants were collected and stored at -80°C until used. The Wnt3a and Wnt5a levels in the supernatants were measured using a mouse Wnt3a and Wnt5a ELISA Kit following the manufacturer’s instructions (Antibodies-online Inc. USA) [22]. For LPS-induced cytokines detection, MSCs were culture in a 96 well plate and treated with or without LPS for 3 days. Then, the concentration of IL-1β, IL-6 and TNF-α in the culture medium was determined by ELISA according to the manufacturer’s instructions (R&D, USA).

### RNA interference

siRNAs specific for mouse Wnt3a (sc-41109), Wnt5a (sc-41113) and control siRNA (sc-36869) with scrambled sequences were purchased from Santa Cruz. In each transfection, 0.1 to 0.2 μM siRNAs were introduced into MSCs using the Lipofectamine 2000 kit (Invitrogen, USA). The efficiency of transduction was determined by detecting the rate of FITC-positive cells and the mRNA expression of specific genes.

### Statistical analysis

All data were collected from three to four independent experiments unless otherwise stated, and were analyzed using the SPSS13.0 software package. A one-way ANOVA followed by Fisher’s post hoc test were used for multiple-group comparisons. Student’s t-test was used when only two sets of data were compared. A statistically significant level was defined as p < 0.05. Error bars are reported as mean ± SEM.

## Results

### TLR4 ligand LPS treatment promotes bone marrow MSC proliferation

**Fig 1:**
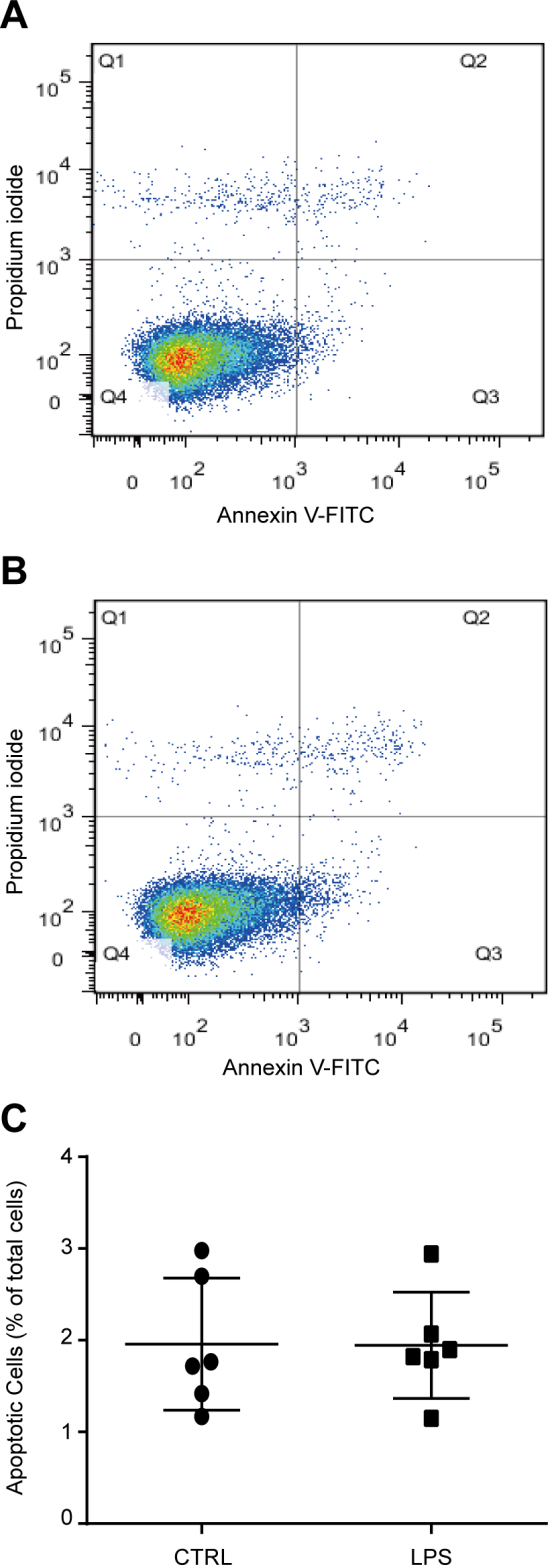
TLR4 ligand LPS treatment did not influence cell apoptosis of bone marrow derived MSCs. MSCs were treated with 1000 ng/ml LPS for 6 days. Total apoptotic cells were detected using flow cytometric analysis with Annexin V and PI staining. **(A, B)** Representative images of control and LPS treated cells. **(C)** Statistical results of six independent experiments.

**Fig 2:**
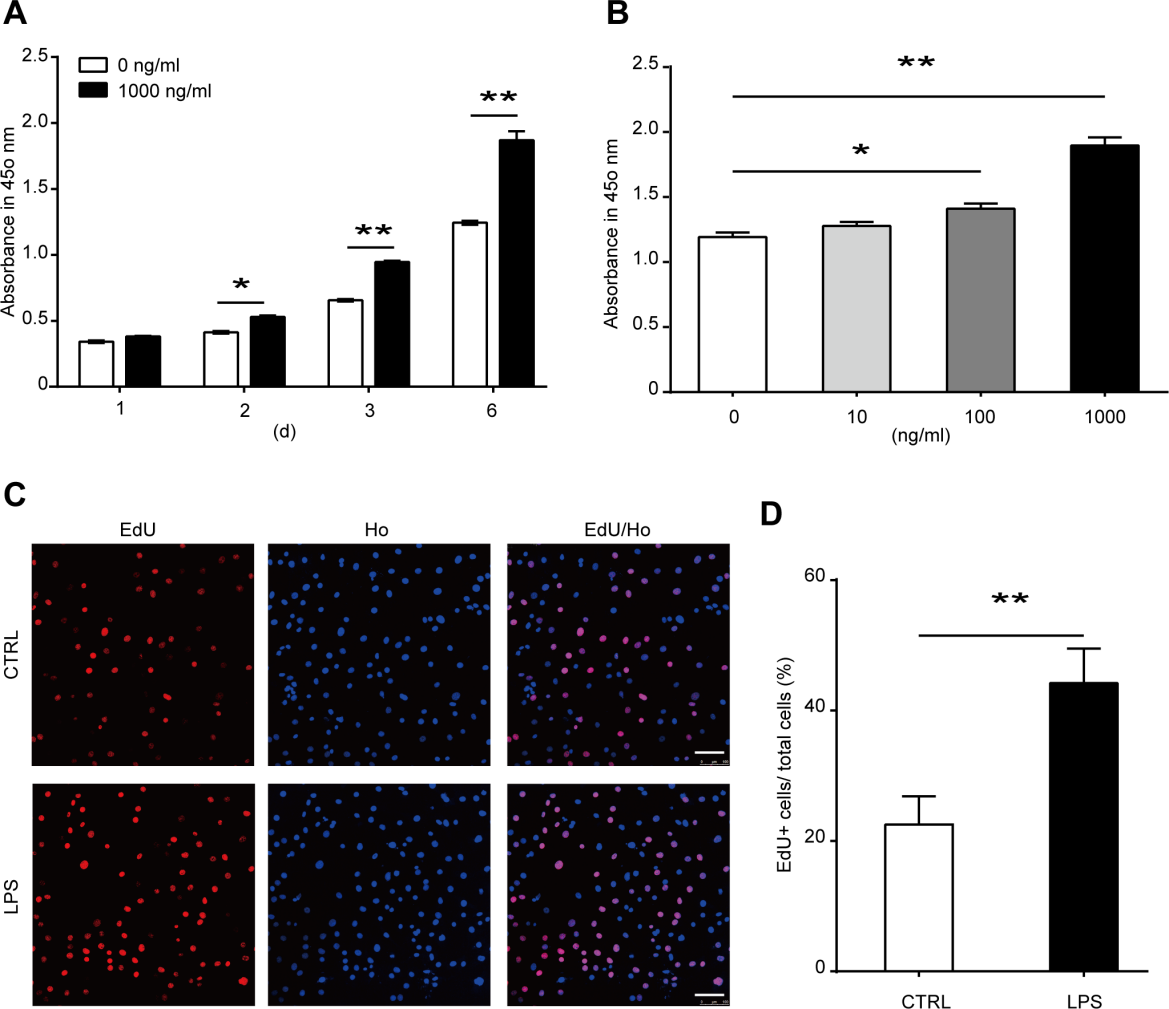
Effects of LPS treatment on the proliferation of MSCs. **(A)** MSCs were treated with 1000 ng/ml LPS for 1, 2, 3, or 6 days. Cell proliferation was detected with CCK-8 kit assay. **(B)** MSCs were treated with various concentrations of LPS. Cell proliferation was examined on the sixth day of treatment with CCK-8 kit assay. **(C, D)** MSCs were treated with 1000 ng/ml LPS for 6 days, and the proliferation of cells was analysis with EdU incorporation. **(C)** The representative images (Scale bar: 100 μm), and **(D)** The statistical result. Data are presented as mean ± SEM from four independent experiments. *P<0.05, **P<0.01.

To study the role of TLR4 in bone marrow MSCs, we firstly cultured MSCs from wild type mouse bone marrow as previously described. The phenotype of the cultured cells was identified using flow cytometric analysis with CD29, CD44, CD31, and CD45 antibodies. Almost all the cells are CD29 and CD44 positive. The cells are also CD31 and CD45 negative (S1 Fig). LPS has been proved to be an effective ligand for TLR4. To activate TLR4, we treated the cells with 1000 ng/ml LPS. The concentration of LPS is widely used in previous studies [31,32]. No change of cell death was induced by 1000 ng/ml LPS as we detected 6 days after treatment (Fig 1A–1C). Then the time-dependent effects and dose-dependent effects of LPS on MSC proliferation was examined using CCK-8 kit assay. MSCs were treated by 1000 ng/ml LPS for 1, 2, 3, and 6 days. LPS treatment significantly promoted MSC proliferation as detected 2 days after treatment, and the effects increased in a time-dependent manner (Fig 2A). Then the cells were treated with 0, 10, 100, and 1000 ng/ml LPS for 6 days. LPS remarkably enhanced MSC proliferation at a concentration of 100 ng/ml, and the effects increased significantly again at a concentration of 1000 ng/ml (Fig 2B). These results were further confirmed by the results from EdU incorporation analysis. We found that the percentage of EdU positive (EdU^+^) cells in LPS treatment group was much higher than control group, indicating the ratio of proliferative cells in LPS treatment group is significantly higher than control group (Fig 2C and 2D). Taken together, these data suggested that TLR4 ligand LPS treatment promoted the proliferation of bone marrow MSCs.

### TLR4 activation by LPS enhances osteogenic differentiation of MSCs

**Fig 3:**
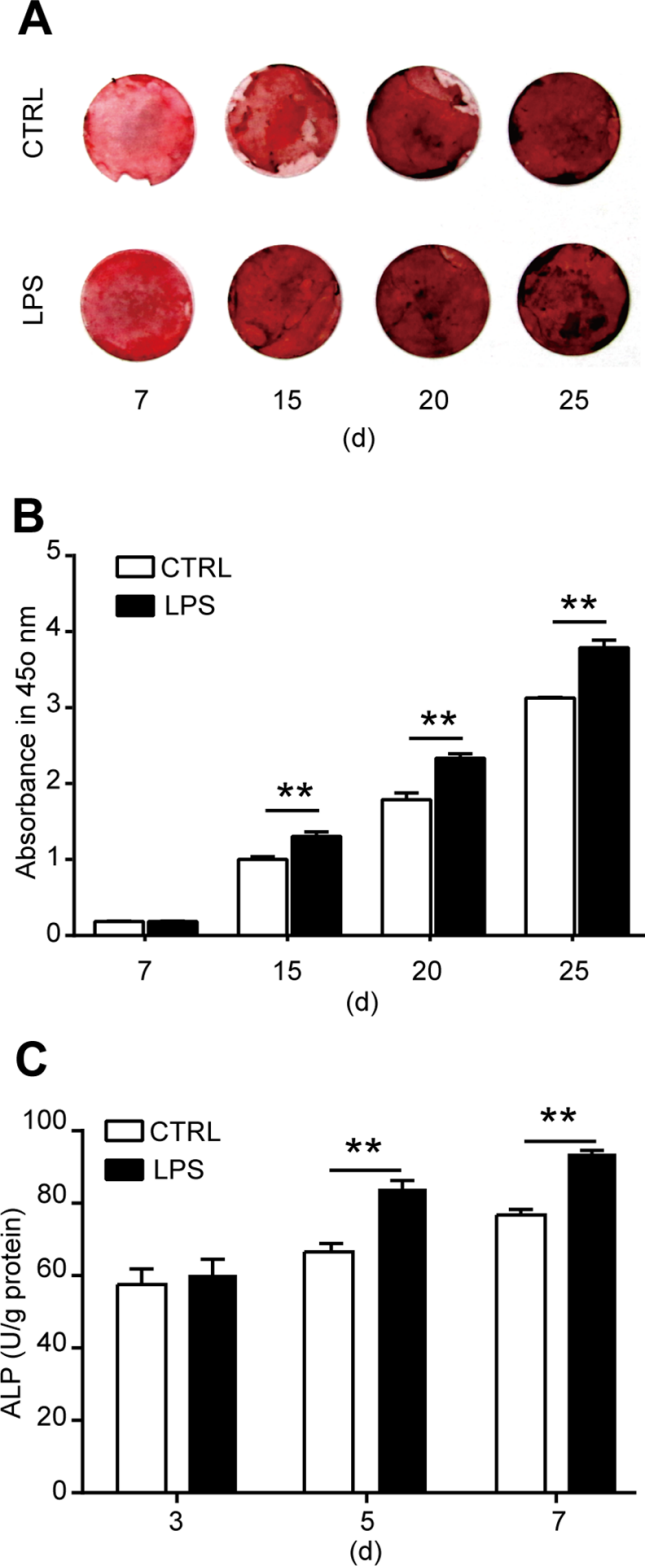
Effects of LPS treatment on the osteogenic differentiation of MSCs. **(A, B)** MSCs were induced for osteogenic differentiation in the presence of 1000 ng/ml LPS as indicated for 7, 15, 20, or 25 days. Alizarin red staining and quantification was used to detect the osteogenic differentiation. **(A)** Representative images. **(B)** Quantification of alizarin red. **(C)** ALP activity after MSCs were induced for osteogenic differentiation in the presence of 1000 ng/ml LPS for 3, 5, or 7 days. Data shown are representative of four independent experiments and presented as mean ± SEM. *P<0.05, **P<0.01.

To reveal the role of TLR4 in the osteogenic differentiation of MSCs, we cultured the cells in an osteogenic differentiated medium. LPS was added to the cultured medium at a final concentration of 1000 ng/ml to activated TLR4. The osteogenic differentiation of MSCs was evaluated by calcium deposit and ALP activity in the differentiated cells. To detect calcium deposit, MSCs were differentiated in the presence of LPS for 7, 15, 20, and 25 days. Alizarin red staining was used to analysis the calcium deposit in the differentiated cells. We found that from the 15th day after LPS treatment, the calcium deposit was remarkably increased as compared with control, and this effect increased in a time-dependent manner (Fig 3A and 3B). Then the ALP activity in the differentiated cells was detected 3, 5, and 7 days after LPS treatment. The ALP activity is enhanced notably at 5 and 7 days after LPS treatment (Fig 3C). From these data, we can get a conclusion that TLR4 activation by its ligand LPS enhanced the osteogenic differentiation of MSCs.

### LPS treatment increases cytokine production in MSCs

**Fig 4:**
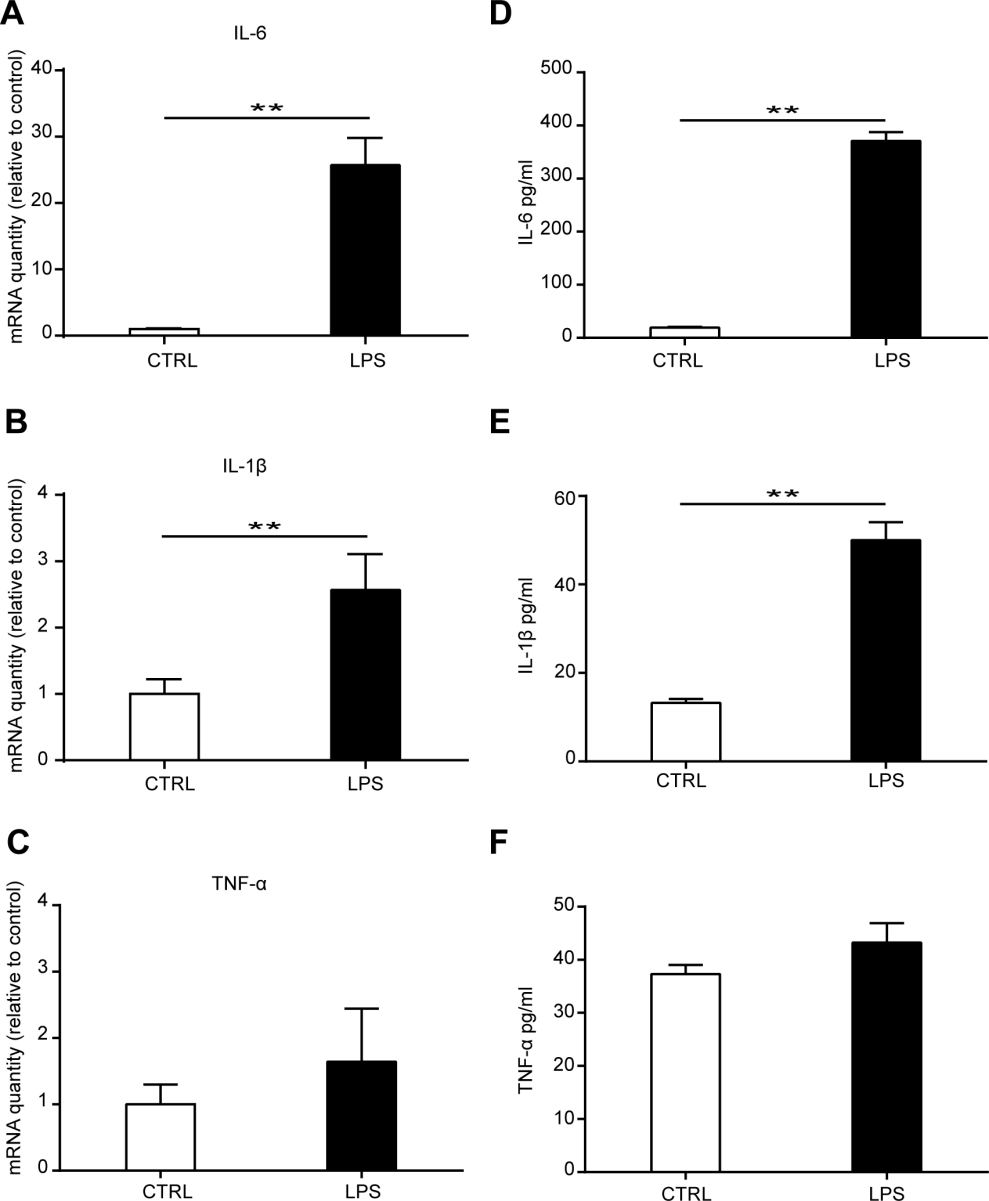
Effects of LPS treatment on cytokine production of MSCs. MSCs were treated with 1000 ng/ml LPS for 3 days. The mRNA production of IL-6 **(A)**, IL-1β **(B)**, and TNF-α **(C)** was detected by Real-time PCR. Data are from four independent experiments and presented as mean ± SEM. **P<0.01. MSCs were cultured in a 96 well plate and treated with 1000 ng/ml LPS for 3 days. The concentration of IL-6 **(D)**, IL-1β **(E)**, and TNF-α **(F)** in the culture medium was detected by ELISA. Data are from three independent experiments and presented as mean ± SEM. **P<0.01.

MSCs have been revealed to be a major source of cytokines in the bone marrow tissues. Some of these cytokines are critical in maintaining the microenvironment for bone tissue regeneration, and plays an important role in stimulating MSC proliferation and differentiation. Cytokines IL-6, IL-1β, and TNF-αplay a critical role in regulating MSC functions as demonstrated in previously researches [33,34,35,36]. To reveal the effects of TLR4 activation on the cytokine production of MSCs, we detected the mRNA and protein expression of IL-6, IL-1β, and TNF-α. We found that the mRNA levels of IL-6 and IL-1β in MSCs were both upregulated after treated with 1000 ng/ml LPS for 3 days. IL-6 mRNA level in LPS treated MSCs was nearly 20 times higher than control (Fig 4A). IL-1β mRNA levels was upregulated by 2.4 times in LPS treated cells compare to control (Fig 4B). Similar results were found in IL-6 and IL-1β protein expression as detected using ELISA 3 days after LPS treatment (Fig 4D and 4E). No significant change of TNF-α mRNA and protein level was found after TLR4 ligand LPS treatment (Fig 4C and 4F). These data suggested that TLR4 activation upregulated cytokines IL-6 and IL-1β production in bone marrow MSCs.

### TLR4 deletion eliminates the effects of LPS on MSC cytokine production, proliferation and osteogenic differentiation

**Fig 5:**
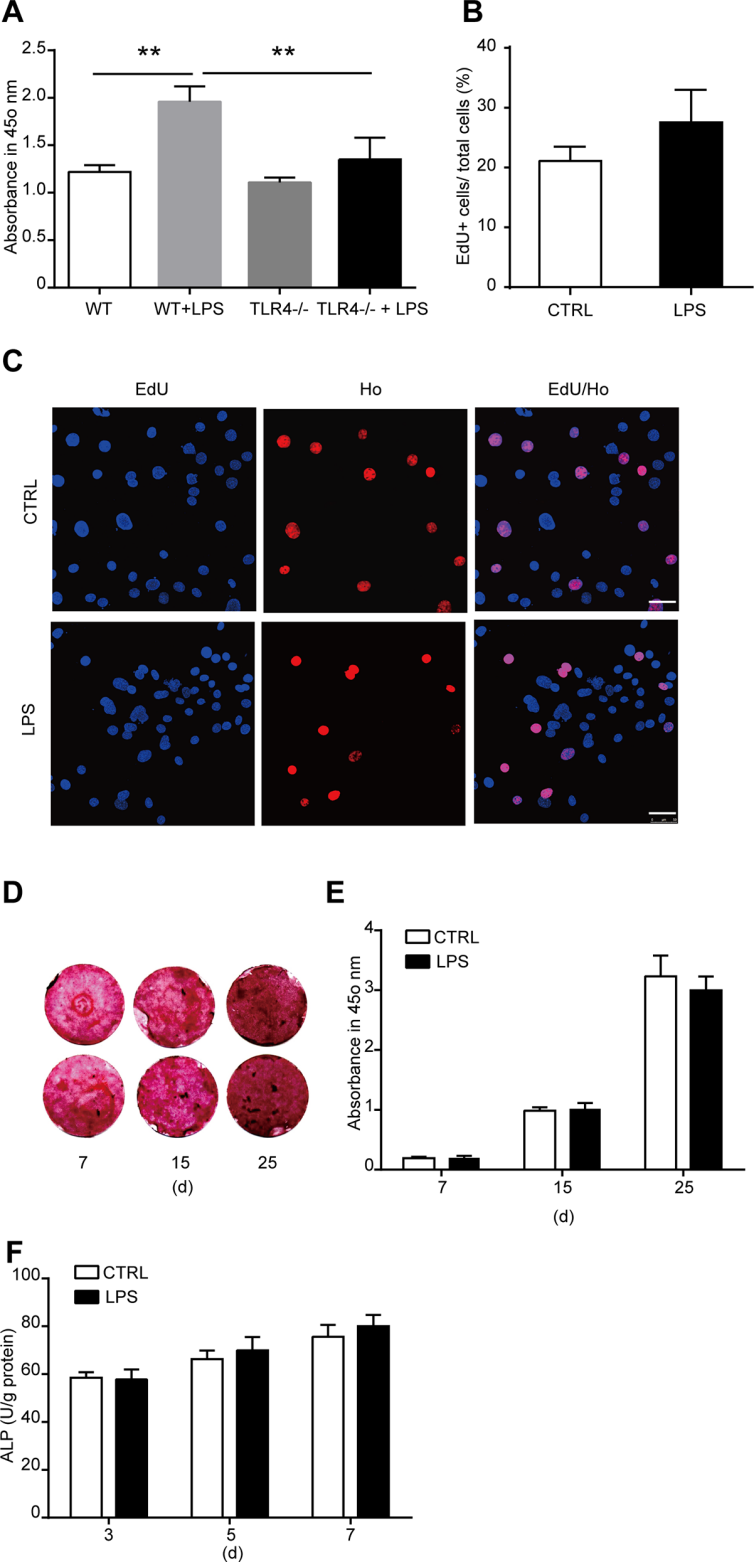
TLR4 knock out eliminate the effects of LPS on MSC proliferation and osteogenic differentiation. **(A)** Wild type (WT) cells and TLR4 knock out (TLR4^-/-^) cells were treated with 1000 ng/ml LPS for 6 days. Cell proliferation was examined by CCK-8 kit assay. **(B, C)** TLR4^-/-^ cells were treated with 1000 ng/ml LPS for 6 days. Cell proliferation was detected by EdU incorporation assay. **(B)** The statistical results. **(C)** Representative images of EdU staining (Scale bar: 50 μm). **(D, E)** MSCs were differentiated with 1000 ng/ml LPS for 7, 15, or 25 days. The osteogenic differentiation was detected by alizarin red staining and quantification. Graph **(D)** shows the representative images, and **(E)** is the quantification results. **(F)** ALP activity of TLR4^-/-^ MSC-differentiated cells in the presence of 1000 ng/ml LPS. Data are presented as mean ± SEM from four independent experiments. **P<0.01.

Previous studied suggested that LPS treatment could modify TLR4 expression [10,37]. Thus, we detected the mRNA and protein expression of TLR4 in MSCs after LPS treatment. We found that TLR4 mRNA and protein level both increased significantly after LPS treatment (S2 Fig). To further reveal the effects of TLR4 on MSC proliferation and osteogenic differentiation, we cultured MSCs from the bone marrow of TLR4^-/-^ mice (S3 Fig). The effects of LPS on MSC cytokine production, proliferation and differentiation were then studied using TLR4^-/-^ MSCs. We found that the effect of LPS on IL-6 and IL-1β production was abolished by TLR4 deletion (S4 Fig). No difference in the cell proliferation of TLR4^-/-^ MSCs was found after LPS treatment for 6 days as detected by CCK-8 kit assay and EdU incorporation (Fig 5A–5C). Furthermore, results from alizarin red staining showed that no change in calcium deposit in the differentiated TLR4^-/-^ cells after LPS treatment was found as detected 7, 15, and 25 days after differentiation. In addition, the ALP activity was not changed after LPS treatment for 3, 5, and 7 days. Together, these results suggested that TLR4 is essential for LPS-induced MSC proliferation, osteogenic differentiation and cytokines production. The results further confirmed the role of TLR4 activation in inducing MSC proliferation and osteogenic differentiation.

### LPS treatment upregulates the expression of Wnt3a and Wnt5a through TLR4

**Fig 6:**
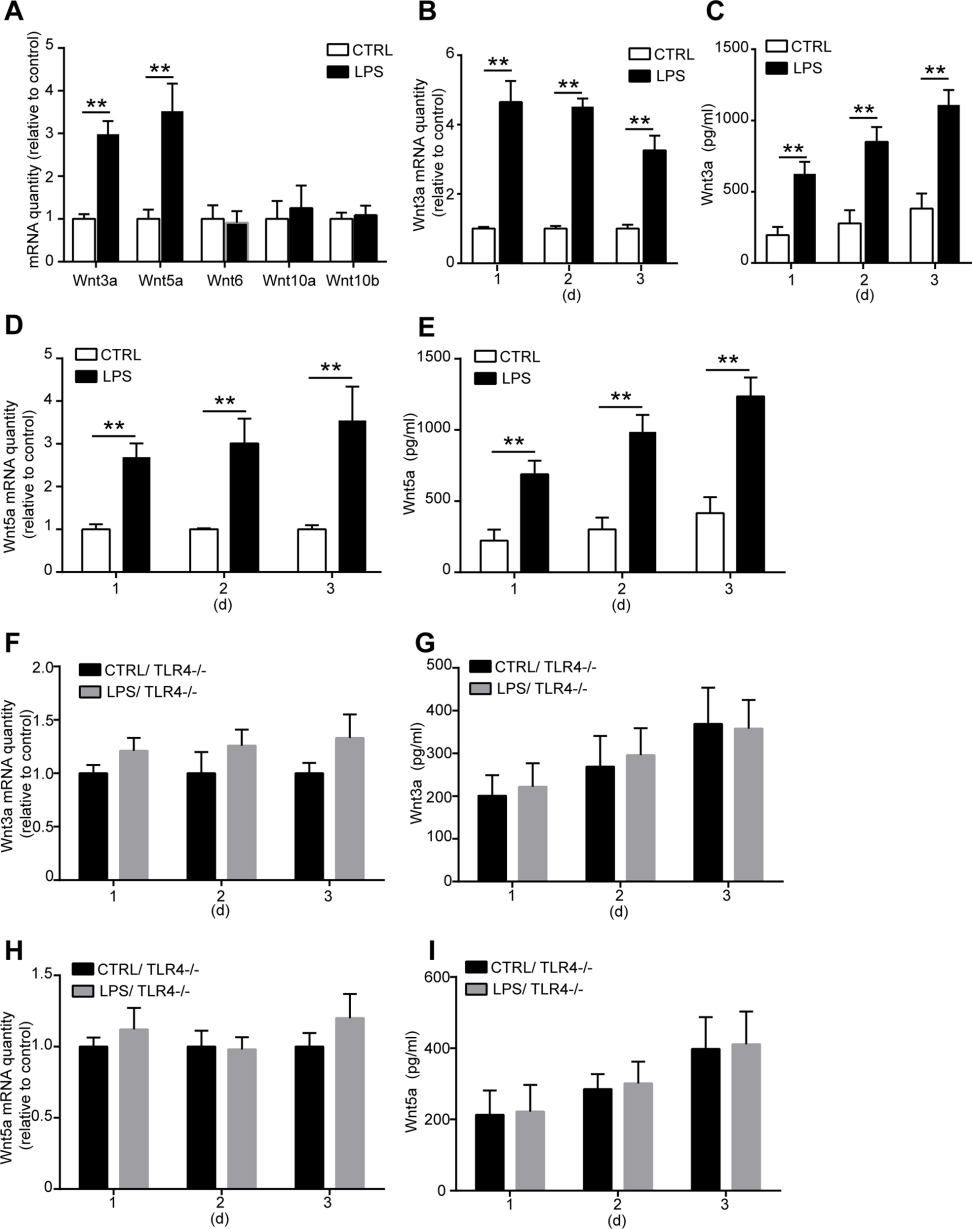
LPS treatment upregulates Wnt3a and Wnt 5a expression through TLR4. **(A)** WT MSCs were treated with 1000 ng/ml LPS for 3 days, the mRNA expression of Wnt family members was detected by real-time PCR. **(B-E)** WT MSCs were treated with 1000 ng/ml LPS for 1, 2, or 3 days, then the mRNA and protein expression of Wnt3a and Wnt5a were detected by real-time PCR and ELISA respectively. **(B)** mRNA expression of Wnt3a in WT MSCs after LPS treatment. **(C)** Protein level of Wnt3a in the culture supernatants after LPS treatment. **(D)** Wnt5a mRNA level in WT MSCs. **(E)** Wnt5a protein concentration in the culture supernatants. **(F-I)** TLR4^-/-^ MSCs were treated with 1000 ng/ml LPS for 1, 2, or 3 days, then the mRNA and protein expression of Wnt3a and Wnt5a were detected as previously described. **(F, G)** Wnt3a mRNA expression in the TLR4^-/-^ MSCs and protein concentration in the culture supernatants. **(H, I)** Wnt5a mRNA level in the TLR4^-/-^ MSCs and protein concentration in the culture supernatants. Data are from four independent experiments and presented as mean ± SEM. **P<0.01.

Wnt signaling is critical in fate decision of stem cell development during the embryonic phase. Recent researches indicate that Wnt signaling also plays an important role in regulating MSC proliferation and differentiation in adult [20,21,24]. To explore the underlying mechanisms of how TLR4 regulates MSC proliferation and osteogenic differentiation, we examined the involvement of Wnt family member. We detected the mRNA levels of Wnt3a, Wnt5a, Wnt6, Wnt10a, and Wnt10b in MSCs after LPS treated for 3 days. Only the mRNA expression of Wnt3a and Wnt5a was significantly increased after TLR4 activation by LPS (Fig 6A). We then analyzed the effects of LPS on the mRNA and protein expression of Wnt3a and Wnt5a. MSCs were treated with 1000 ng/ml LPS for 1, 2, and 3 days. Wnt3a and Wnt5a mRNA level in MSCs and the protein concentration in the culture supernatants were examined. We found that both Wnt3a and Wnt5a mRNA level were upregulated as detected in all the 3 days after LPS treatment (Fig 6B and 6C). Furthermore, the protein concentration of Wnt3a and Wnt5a in the culture medium was also increased in a time-dependent manner as detected by ELISA (Fig 6D and 6E). To further confirm the relationship among Wnt3a, Wnt5a, and TLR4, the TLR4^-/-^ MSCs was used in the experiments. No change of Wnt3a and Wnt5a mRNA expressions was found after LPS treatment in TLR4^-/-^ MSCs (Fig 6F and 6H). In addition, no increase of the protein concentrations of Wnt3a and Wnt5a was found in the culture supernatants of TLR4^-/-^ MSCs (Fig 6G and 6I). These results strongly indicated that TLR4 is essential for LPS-induced Wnt3a and Wnt5a upregulation.

### Wnt3a plays a critical role for TLR4-induced MSC proliferation, while Wnt5a is involved in TLR4-induced MSC osteogenic differentiation

**Fig 7:**
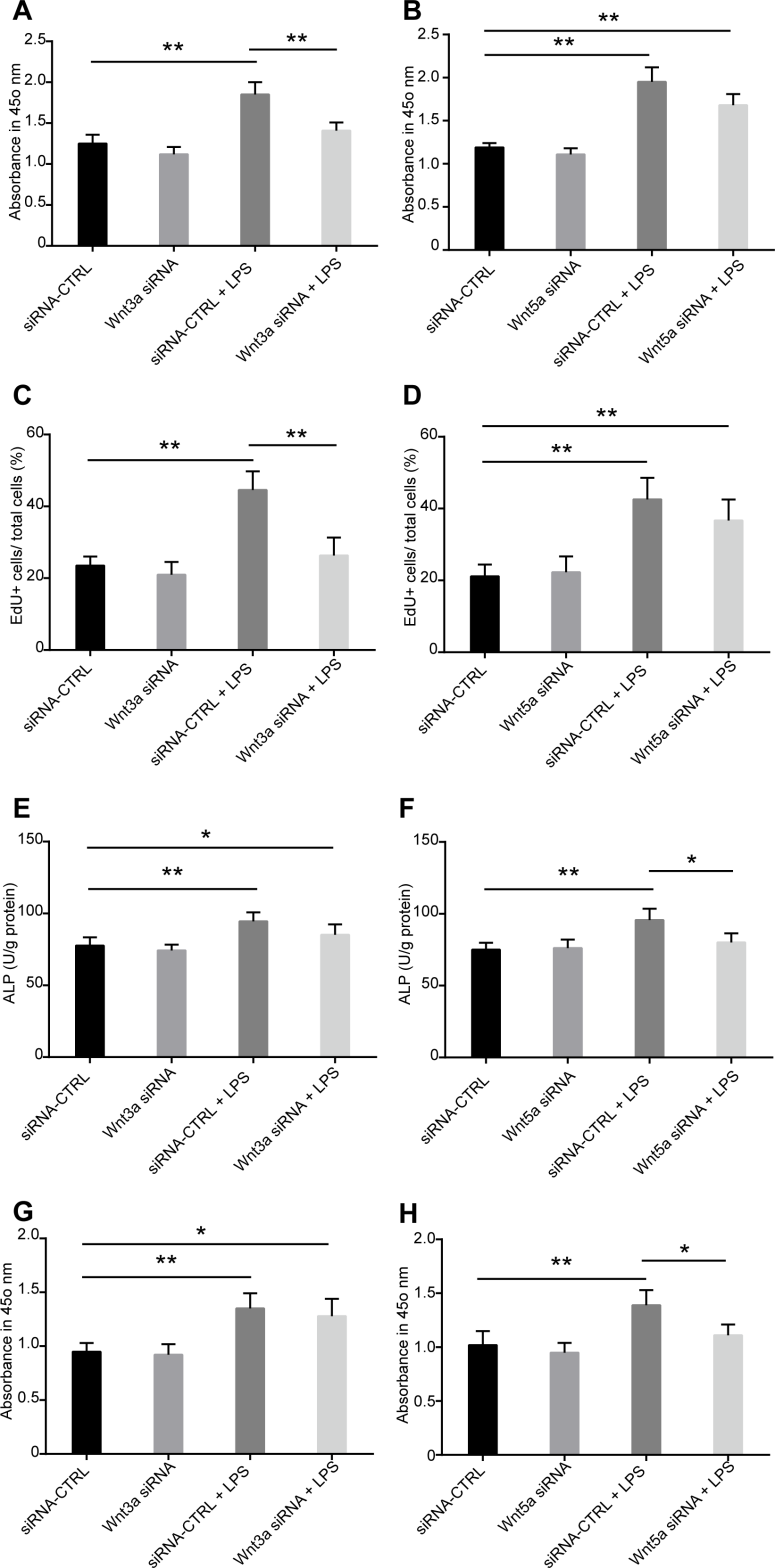
Proliferation and osteogenic differentiation of MSCs in the presence of LPS after Wnt3a and Wnt5a silence. **(A-D)** MSCs were treated by Wnt3a and Wnt5a siRNA. The effect of LPS on MSC proliferation was detected using CCK-8 kit assay and EdU incorporation. **(A, B)** MSCs were transfected with Wnt3a and Wnt5a siRNA, then the cells were treated with 1000 ng/ml LPS for 6 days. Cell proliferation was detected by CCK-8 kit assay. **(C, D)** Statistical results of cell proliferation from EdU incorporation as detected 6 days after LPS treatment. Representative images see S6 Fig. **(E-H)** Wnt3a and Wnt5a expression were silenced by specific siRNA, then the cells were differentiated in the presence of 1000 ng/ml LPS. ALP activity and alizarin red staining was used to analyzed the effects of LPS on osteogenic differentiation of MSCs. **(E, F)** ALP activity in MSC-differentiated cells 7 days after LPS treatment. **(G, H)** Quantification of alizarin red staining in MSC-differentiated cells 15 days after LPS treatment. Representative images of alizarin red staining see S6 Fig. Data are from three independent experiments and presented as mean ± SEM. *P<0.05, **P<0.01.

To explore the role of Wnt3a and Wnt5a in TLR4-induced MSC proliferation and osteogenic differentiation, specific siRNAs were used to silence Wnt3a and Wnt5a expression before LPS treatment (S5A and S5B Fig). We found that Wnt3a and Wnt5a silence has little effect on LPS-induced IL-6 and IL-1β secretion (S5C–S5F Fig), which indicated that Wnt3a and Wnt5a are not involved in TLR4-induced cytokine production. The results from CCK-8 kit assay suggested that Wnt3a silencing remarkably inhibited the effects of LPS on MSC proliferation (Fig 7A). However, Wnt5a siRNA treatment has little effect on eliminating the effects of LPS on MSC proliferation (Fig 7B). The result was further confirmed by EdU incorporation assay (Fig 7C and 7D and S6A and S6B Fig). These results indicated that Wnt3a is critical for TLR4-induced MSC proliferation, while Wnt5a plays little effect in this process. Followed, the effects of LPS on the ALP activity in differentiated cells was detected after Wnt3a and Wnt5a specific siRNAs transfection. We found that Wnt3a siRNA treatment has little influence on changing the effects of LPS on ALP activity (Fig 7E). Wnt5a silence significantly antagonized the effects of LPS on increasing ALP activity (Fig 7F). Furthermore, the same role of Wnt3a and Wnt5a siRNAs was found in influencing the calcium deposit in the differentiated cells. We found that Wnt5a siRNA treatment significantly antagonized the effects of LPS on calcium deposit, while Wnt3a siRNA has little effect on this process (Fig 7G and 7H and S6C and S6D Fig). These results suggested that Wnt5a is involved in TLR4-induced MSC osteogenic differentiation, while Wnt3a plays little effect in this process.

## Discussion

TLR4 plays an important role in regulating MSC function. However, the exact functional roles and mechanisms of TLR4 on bone marrow MSC proliferation and differentiation need further study. Here, we found that activation of TLR4 enhances MSC proliferation and osteogenic differentiation. In addition, we also revealed that Wnt3a mediated the effects of TLR4-induced MSC proliferation, while Wnt5a is essential for the promotive effects of TLR4 on MSC osteogenic differentiation.

MSCs from bone marrow give rise to bone tissue. It is the basic cellular unit of embryonic bone formation. MSCs also play a key role in fracture repair by differentiating into bone-forming osteoblasts. Many exciting findings have been obtained in resent MSC transplantation experiments in animal models. The enhancement of bone regeneration with MSCs is becoming a clinical reality [7]. Among all types of the adult stem cells, MSCs are the easiest to isolate, and are multipotent. In addition, MSCs can been easily expanded *in vitro* [38]. Thus, MSCs serve as the best candidate cells for tissue engineering. To promote the application of MSCs in bone regeneration and tissue engineering, an understanding of MSC biology is necessary.

MSC osteogenic differentiation is controlling by the coordinate activities of different signaling pathways that regulate the expression of various osteoblast-specific genes. Recently, TLR4 signaling is found expressed in bone marrow derived MSCs and play a critical role in regulating the function of MSCs [11]. Under pathological conditions, such as ischemia, traumatic injury, inflammatory process, endogenous and exogenous bacteria could release TLR4 ligand LPS which will activate TLR4 signaling in many different type of cells [39,40]. How MSCs maintain their multipotency and capacity to self-renewal and differentiate in these pathological conditions is not well understood. This is an important question for the further application of MSCs in the clinic.

Our results shown that, TLR4 activation by LPS promoted MSC proliferation and osteogenic differentiation *in vitro*. Previous study has revealed that LPS is able to induce proliferation and osteogenic differentiation but reduce adipogenic differentiation of human adipose tissue-derived MSCs *in vitro* [15]. However, the tissue origin of MSCs influences their TLR profile as well as their functional properties such as cytokine secretion, proliferation and osteogenic potential [14,16]. The reason may due to the divergent levels of several important signaling pathways which mediate TLR4 functions, such as LBP and TGFβ1 signaling [41]. Our results provide evidence that TLR4 signaling plays an important role in regulating bone marrow derived MSC function. Understanding the role and mechanisms of TLR4 on MSC proliferation and differentiation may shed light on the bone remodeling. More importantly, it is also critical for development of new therapy using MSCs under pathological condition by modulation of TLR4 signaling activity.

It has become clear that MSCs also possess immunoregulatory properties during the past few years [5,6]. We have detected the cytokine production in MSCs after TLR4 activation. We found that IL-1β and IL-6 production in MSCs is upregulated after treated by TLR4 ligand LPS. However, we did not find any changes in cell survival, indicating the production of these cytokines does not kill the cells. Cytokines, IL-1β and IL-6 etc., induced by LPS may have the function of enhance TLR4 expression in MSCs [10,37]. Our result confirmed that as proved by the upregulation of TLR4 expression after LPS treatment. These results suggested that upregulating TLR4 expressing may be another mechanism by which LPS modulating TLR4 signaling activity in MSCs. IL-1β and IL-6 can also induce MSCs migration by inducing the expression of chemokines [36]. Thus, the effects of LPS on cytokine secretion probably will enhance the function of TLR4 on bone tissue generation from MSCs. It is found that prolonged exposure to bacterial toxins downregulated expression of TLR4 in MSC-derived osteoprogenitors [42]. High concentration of LPS was found to reduce MSC proliferation, osteogenic differentiation, and increase cell death [43]. These results suggested that the time phase and dose of TLR4 ligands treatment are also key factors in regulating MSC functions.

Exploring the molecular mechanisms of TLR4 on MSC functions is critical for developing new therapy under pathological condition by modulating TLR4 activity. Till now, how TLR4 activation regulates MSC proliferation and differentiation is unclear. Published data suggest that Wnt signaling is critical in determining both embryonic and adult MSC fate. Wnt6, Wnt10a and Wnt10b are found to inhibit adipogenesis and stimulate osteoblastogenesis through a β-catenin-dependent mechanism [25]. Wnt3a signaling promotes the proliferation, myogenic differentiation, and migration of bone marrow derived MSCs [20,21]. Wnt5a is necessary to maintain osteogenic potential of human MSCs, and it is also found to mediate the effects of the LIM-only protein FHL2 on osteogenic differentiation of MSCs [23,24]. However, the interactions of TLR4 and Wnt signaling and their functions in regulating the proliferation and differentiation of MSCs are unknown. We screened the mRNA expressions of Wnt family members Wnt3a, Wnt5a, Wnt6, Wnt10a, and Wnt10b in response to TLR4 ligand LPS treatment. We found that Wnt3a and Wnt5a mRNA and protein levels are significantly upregulated after TLR4 activation by LPS. Further studies using Wnt3a and Wnt5a specific siRNA revealed that Wnt3a is critical for TLR4-induced MSC proliferation. Wnt5a is essential for mediating the effects of TLR4 on MSC osteogenic differentiation. Our data revealed the interactions between TLR4 and Wnt signaling member Wnt3a and Wnt5a, and the functional role of this interaction in regulating MSC proliferation and osteogenic differentiation. It is found that LPS enhances Wnt5a expression through TLR4, myeloid differentiating factor 88, phosphatidylinositol 3-OH kinase/AKT and nuclear factor Kappa B pathways in human dental pulp stem cells [22]. TLR4 was also reported to downregulate the Wnt pathway in enterocytes in the ileum of newborn mice [44]. These results raise the possibility that TLR4 may play diverse role in regulating Wnt signaling in different tissues. Our data revealed that both Wnt3a and Wnt5a play critical, but different, roles in TLR4-induced MSC proliferation and osteogenic differentiation. However, Wnt3a and Wnt5a silence has little effect on the LPS-induced cytokines production. Probably other mechanisms exist in mediating the effects of TLR4-induced cytokine secretion in MSCs. We did not analysis the dynamic changes and functions of all the Wnt family members during the whole process of MSC migration, proliferation, osteogenic differentiation, and maturation. In addition, we could not exclude that other Wnt family members is involved in MSC proliferation and osteogenic differentiation only depending on the results of their expression in this process. Further researches are still needed to further exploring the dynamic role of different Wnt family members in controlling the MSC fate and their interaction with TLR4. To do this, the specific Wnt-CreER recombinase transgenic mice are probably needed to conditionally delete the Wnt expression at different stages to determine the role of Wnt signaling *in vivo*.

In conclusion, we have revealed the regulation of TLR4 on Wnt signaling in bone marrow derived MSCs and their function in determining the proliferation and differentiation of MSCs. These finding provide new data to understand the role of TLR4 in regulating MSC fates and the underlying molecular mechanisms. These data also provide new insight in developing new therapy using MSCs by modulating TLR4 signaling activity.

## Supporting Information

S1 Fig Identification of bone marrow derived MSCs from wild type mice. The flow cytometric analysis shows that the cultured cells are CD29 **(B)** and CD44 **(C)** positive. The cells are also CD31 **(D)** and CD45 **(E)** negative.
(TIF)

S2 Fig LPS treatment increases TLR4 expression in MSCs. **(A)** The mRNA expression of TLR4 in MSCs was upregulated after 1000 ng/ml LPS treated for 3 days as detected by real-time PCR analysis. **(B, C)** The protein level of TLR4 is increased by LPS treatment as detected by western blotting analysis. Data are from three independent experiments and presented as mean ± SEM. *P<0.05, **P<0.01.
(TIF)

S3 Fig (A) PCR identification of TLR4 KO mice. Genomic DNAs were extracted from tails of the mouse and were analyzed by PCR using the following primers: wild-type TLR4 primer (5'ATATGCATGATCAACACCACAG 3' and 5' TTTCCATTGCTGCCCTATAG 3'), mutant TLR4 primer (5'GCAAGTTTCTATATGCATTCTC 3' and 5' CCTCCATTTCCAATAGGTAG 3'). The 140 bp band is the mutant TLR4 (lane 2 to 4), while the 390 bp band represented the wild-type genotype of TLR4 (lane 5 to 7). (B-E) Characteristic of bone marrow derived MSCs from TLR4 KO mice. The cultured cells are CD29^+^ **(B)**, CD44^+^ **(C)**, CD31 ^-^**(D)**, and CD45 ^**-**^**(E)**.
(TIF)

S4 Fig TLR4 deletion eliminates the effect of LPS on MSC cytokine production. TLR4-/- MSCs were treated by 1000 ng/ml LPS for 3 days, the mRNA ecpression of IL-1β **(A)** and IL-6 **(B)** were detected by real-time PCR. No change was found in both of IL-1β and IL-6 mRNA expression. Data are from three independent experiments and presented as mean ± SEM.
(TIF)

S5 Fig **(A, B)** Wnt3a and Wnt5a siRNA transfection effectively silence Wnt3a and Wnt5a expression. MSCs were transfected with Wnt3a and Wnt5a siRNA respectively. The mRNA expression of Wnt3a **(A)** and Wnt5a **(B)** was detected by real-time PCR 2 and 4 days after transfection. Data are from three independent experiments and presented as mean ± SEM. **P<0.01. (C-F) Effects of Wnt3a and Wnt5a silence on LPS-induced cytokine production in MSCs. Wild type MSCs were transfected with Wnt3a (C, D) or Wnt5a (E, F) siRNA respectively, the mRNA expression of IL-1β and IL-6 was then detected by real-time PCR. Data are from three independent experiments and presented as mean ± SEM. **P<0.01.
(TIF)

S6 Fig **(A, B)** Representative images of EdU incorporation as detected 6 days after LPS treatment in Wnt3a and Wnt5a silence cells. Scale bar: 50 μm. **(C, D)** Representative images of alizarin red staining as detected 15 days after LPS treatment. Scale bar: 100 μm.
(TIF)

## References

[1] Pittenger MF, Mackay AM, Beck SC, Jaiswal RK, Douglas R, Mosca JD, et al Multilineage potential of adult human mesenchymal stem cells. Science. 1999;284(5411):143–7. Epub 1999/04/02. . 10102814 10.1126/science.284.5411.143

[2] Gronthos S, Zannettino AC, Hay SJ, Shi S, Graves SE, Kortesidis A, et al Molecular and cellular characterisation of highly purified stromal stem cells derived from human bone marrow. J Cell Sci. 2003;116(Pt 9):1827–35. Epub 2003/04/01. . 12665563 10.1242/jcs.00369

[3] Bruder SP, Kurth AA, Shea M, Hayes WC, Jaiswal N, Kadiyala S. Bone regeneration by implantation of purified, culture-expanded human mesenchymal stem cells. J Orthop Res. 1998;16(2):155–62. Epub 1998/06/11. 10.1002/jor.1100160202 . 9621889

[4] Livingston TL, Gordon S, Archambault M, Kadiyala S, McIntosh K, Smith A, et al Mesenchymal stem cells combined with biphasic calcium phosphate ceramics promote bone regeneration. J Mater Sci Mater Med. 2003;14(3):211–8. Epub 2004/09/07. 5119192 [pii]. . 15348466 10.1023/a:1022824505404

[5] Glenn JD, Whartenby KA. Mesenchymal stem cells: Emerging mechanisms of immunomodulation and therapy. World J Stem Cells. 2014;6(5):526–39. Epub 2014/11/27. 10.4252/wjsc.v6.i5.526 25426250 PMC4178253

[6] Glennie S, Soeiro I, Dyson PJ, Lam EW, Dazzi F. Bone marrow mesenchymal stem cells induce division arrest anergy of activated T cells. Blood. 2005;105(7):2821–7. Epub 2004/12/14. 2004-09-3696 [pii]. 10.1182/blood-2004-09-3696 . 15591115

[7] Jones E, Yang X. Mesenchymal stem cells and bone regeneration: current status. Injury. 2011;42(6):562–8. Epub 2011/04/15. 10.1016/j.injury.2011.03.030 S0020-1383(11)00124-0 [pii]. . 21489533

[8] Akira S, Takeda K, Kaisho T. Toll-like receptors: critical proteins linking innate and acquired immunity. Nat Immunol. 2001;2(8):675–80. Epub 2001/07/31. doi: 10.1038/90609. 90609 [pii]. . 11477402 10.1038/90609

[9] Medzhitov R. Toll-like receptors and innate immunity. Nat Rev Immunol. 2001;1(2):135–45. Epub 2002/03/22. 10.1038/35100529 . 11905821

[10] Raicevic G, Rouas R, Najar M, Stordeur P, Boufker HI, Bron D, et al Inflammation modifies the pattern and the function of Toll-like receptors expressed by human mesenchymal stromal cells. Hum Immunol. 2010;71(3):235–44. Epub 2009/12/26. 10.1016/j.humimm.2009.12.005 S0198-8859(09)00659-4 [pii]. . 20034529

[11] Liotta F, Angeli R, Cosmi L, Fili L, Manuelli C, Frosali F, et al Toll-like receptors 3 and 4 are expressed by human bone marrow-derived mesenchymal stem cells and can inhibit their T-cell modulatory activity by impairing Notch signaling. Stem Cells. 2008;26(1):279–89. Epub 2007/10/27. 2007–0454 [pii]. 10.1634/stemcells.2007-0454 . 17962701

[12] Wang ZJ, Zhang FM, Wang LS, Yao YW, Zhao Q, Gao X. Lipopolysaccharides can protect mesenchymal stem cells (MSCs) from oxidative stress-induced apoptosis and enhance proliferation of MSCs via Toll-like receptor(TLR)-4 and PI3K/Akt. Cell Biol Int. 2009;33(6):665–74. Epub 2009/04/21. 10.1016/j.cellbi.2009.03.006 S1065-6995(09)00075-4 [pii]. . 19376254

[13] Brewster BD, Rouch JD, Wang M, Meldrum DR. Toll-like receptor 4 ablation improves stem cell survival after hypoxic injury. J Surg Res. 2012;177(2):330–3. Epub 2012/06/19. 10.1016/j.jss.2012.04.042 S0022-4804(12)00402-7 [pii]. . 22703984

[14] Raicevic G, Najar M, Pieters K, De Bruyn C, Meuleman N, Bron D, et al Inflammation and Toll-like receptor ligation differentially affect the osteogenic potential of human mesenchymal stromal cells depending on their tissue origin. Tissue Eng Part A. 2012;18(13–14):1410–8. Epub 2012/03/21. 10.1089/ten.TEA.2011.0434 . 22429150

[15] Fiedler T, Salamon A, Adam S, Herzmann N, Taubenheim J, Peters K. Impact of bacteria and bacterial components on osteogenic and adipogenic differentiation of adipose-derived mesenchymal stem cells. Exp Cell Res. 2013;319(18):2883–92. Epub 2013/08/31. 10.1016/j.yexcr.2013.08.020 S0014-4827(13)00363-7 [pii]. . 23988607

[16] Raicevic G, Najar M, Stamatopoulos B, De Bruyn C, Meuleman N, Bron D, et al The source of human mesenchymal stromal cells influences their TLR profile as well as their functional properties. Cell Immunol. 2011;270(2):207–16. Epub 2011/06/28. 10.1016/j.cellimm.2011.05.010 S0008-8749(11)00120-1 [pii]. . 21700275

[17] Moon RT, Kohn AD, De Ferrari GV, Kaykas A. WNT and beta-catenin signalling: diseases and therapies. Nat Rev Genet. 2004;5(9):691–701. Epub 2004/09/17. 10.1038/nrg1427 nrg1427 [pii]. . 15372092

[18] Yu JM, Kim JH, Song GS, Jung JS. Increase in proliferation and differentiation of neural progenitor cells isolated from postnatal and adult mice brain by Wnt-3a and Wnt-5a. Mol Cell Biochem. 2006;288(1–2):17–28. Epub 2006/04/04. 10.1007/s11010-005-9113-3 . 16583142

[19] Reya T, Clevers H. Wnt signalling in stem cells and cancer. Nature. 2005;434(7035):843–50. Epub 2005/04/15. nature03319 [pii]. 10.1038/nature03319 . 15829953

[20] Boland GM, Perkins G, Hall DJ, Tuan RS. Wnt 3a promotes proliferation and suppresses osteogenic differentiation of adult human mesenchymal stem cells. J Cell Biochem. 2004;93(6):1210–30. Epub 2004/10/16. 10.1002/jcb.20284 . 15486964

[21] Shang YC, Wang SH, Xiong F, Zhao CP, Peng FN, Feng SW, et al Wnt3a signaling promotes proliferation, myogenic differentiation, and migration of rat bone marrow mesenchymal stem cells. Acta Pharmacol Sin. 2007;28(11):1761–74. Epub 2007/10/26. 10.1111/j.1745-7254.2007.00671.x . 17959027

[22] He W, Wang Z, Zhou Z, Zhang Y, Zhu Q, Wei K, et al Lipopolysaccharide enhances Wnt5a expression through toll-like receptor 4, myeloid differentiating factor 88, phosphatidylinositol 3-OH kinase/AKT and nuclear factor kappa B pathways in human dental pulp stem cells. J Endod. 2014;40(1):69–75. Epub 2013/12/18. 10.1016/j.joen.2013.09.011 S0099-2399(13)00818-2 [pii]. . 24331994

[23] Bilkovski R, Schulte DM, Oberhauser F, Gomolka M, Udelhoven M, Hettich MM, et al Role of WNT-5a in the determination of human mesenchymal stem cells into preadipocytes. J Biol Chem. 2010;285(9):6170–8. Epub 2009/12/25. 10.1074/jbc.M109.054338 M109.054338 [pii]. 20032469 PMC2825412

[24] Brun J, Fromigue O, Dieudonne FX, Marty C, Chen J, Dahan J, et al The LIM-only protein FHL2 controls mesenchymal cell osteogenic differentiation and bone formation through Wnt5a and Wnt10b. Bone. 2013;53(1):6–12. Epub 2012/12/04. 10.1016/j.bone.2012.11.020 S8756-3282(12)01382-8 [pii]. . 23201222

[25] Cawthorn WP, Bree AJ, Yao Y, Du B, Hemati N, Martinez-Santibanez G, et al Wnt6, Wnt10a and Wnt10b inhibit adipogenesis and stimulate osteoblastogenesis through a beta-catenin-dependent mechanism. Bone. 2012;50(2):477–89. Epub 2011/08/30. 10.1016/j.bone.2011.08.010 S8756-3282(11)01153-7 [pii]. 21872687 PMC3261372

[26] Chen Q, Shou P, Zhang L, Xu C, Zheng C, Han Y, et al An osteopontin-integrin interaction plays a critical role in directing adipogenesis and osteogenesis by mesenchymal stem cells. Stem Cells. 2014;32(2):327–37. Epub 2013/10/15. 10.1002/stem.1567 24123709 PMC3961005

[27] Xu N, Liu H, Qu F, Fan J, Mao K, Yin Y, et al Hypoxia inhibits the differentiation of mesenchymal stem cells into osteoblasts by activation of Notch signaling. Exp Mol Pathol. 2013;94(1):33–9. Epub 2012/09/12. 10.1016/j.yexmp.2012.08.003 S0014-4800(12)00117-7 [pii]. . 22964414

[28] Chen C, Ma Q, Chen X, Zhong M, Deng P, Zhu G, et al Thyroid Hormone-Otx2 Signaling Is Required for Embryonic Ventral Midbrain Neural Stem Cells Differentiated into Dopamine Neurons. Stem Cells Dev. 2015;24(15):1751–65. Epub 2015/04/14. 10.1089/scd.2014.0489 25867707 PMC4507356

[29] Fu YC, Lin CC, Chang JK, Chen CH, Tai IC, Wang GJ, et al A novel single pulsed electromagnetic field stimulates osteogenesis of bone marrow mesenchymal stem cells and bone repair. PLoS One. 2014;9(3):e91581 Epub 2014/03/19. 10.1371/journal.pone.0091581 PONE-D-13-39187 [pii]. 24632682 PMC3954729

[30] Chen C, Zhou Z, Zhong M, Li M, Yang X, Zhang Y, et al Excess thyroid hormone inhibits embryonic neural stem/progenitor cells proliferation and maintenance through STAT3 signalling pathway. Neurotox Res. 2011;20(1):15–25. Epub 2010/08/17. 10.1007/s12640-010-9214-y . 20711698

[31] Gao S, Mao F, Zhang B, Zhang L, Zhang X, Wang M, et al Mouse bone marrow-derived mesenchymal stem cells induce macrophage M2 polarization through the nuclear factor-kappaB and signal transducer and activator of transcription 3 pathways. Exp Biol Med (Maywood). 2014;239(3):366–75. Epub 2014/02/07. 10.1177/1535370213518169 1535370213518169 [pii]. . 24500984

[32] Rahmat Z, Jose S, Ramasamy R, Vidyadaran S. Reciprocal interactions of mouse bone marrow-derived mesenchymal stem cells and BV2 microglia after lipopolysaccharide stimulation. Stem Cell Res Ther. 2013;4(1):12 Epub 2013/01/30. 10.1186/scrt160 scrt160 [pii]. 23356521 PMC3706938

[33] Ebert R, Benisch P, Krug M, Zeck S, Meissner-Weigl J, Steinert A, et al Acute phase serum amyloid A induces proinflammatory cytokines and mineralization via toll-like receptor 4 in mesenchymal stem cells. Stem Cell Res. 2015;15(1):231–9. Epub 2015/07/03. 10.1016/j.scr.2015.06.008 S1873-5061(15)00081-1 [pii]. . 26135899

[34] Hwang SH, Cho HK, Park SH, Lee W, Lee HJ, Lee DC, et al Toll like receptor 3 & 4 responses of human turbinate derived mesenchymal stem cells: stimulation by double stranded RNA and lipopolysaccharide. PLoS One. 2014;9(7):e101558 Epub 2014/07/09. 10.1371/journal.pone.0101558 PONE-D-14-08939 [pii]. 25004159 PMC4086816

[35] Kondo M, Yamaoka K, Sakata K, Sonomoto K, Lin L, Nakano K, et al Contribution of the Interleukin-6/STAT-3 Signaling Pathway to Chondrogenic Differentiation of Human Mesenchymal Stem Cells. Arthritis Rheumatol. 2015;67(5):1250–60. Epub 2015/01/22. 10.1002/art.39036 . 25604648

[36] Carrero R, Cerrada I, Lledo E, Dopazo J, Garcia-Garcia F, Rubio MP, et al IL1beta induces mesenchymal stem cells migration and leucocyte chemotaxis through NF-kappaB. Stem Cell Rev. 2012;8(3):905–16. Epub 2012/04/03. 10.1007/s12015-012-9364-9 22467443 PMC3412085

[37] Romieu-Mourez R, Francois M, Boivin MN, Bouchentouf M, Spaner DE, Galipeau J. Cytokine modulation of TLR expression and activation in mesenchymal stromal cells leads to a proinflammatory phenotype. J Immunol. 2009;182(12):7963–73. Epub 2009/06/06. 10.4049/jimmunol.0803864 182/12/7963 [pii]. . 19494321

[38] Satija NK, Gurudutta GU, Sharma S, Afrin F, Gupta P, Verma YK, et al Mesenchymal stem cells: molecular targets for tissue engineering. Stem Cells Dev. 2007;16(1):7–23. Epub 2007/03/14. 10.1089/scd.2006.9998 . 17348802

[39] Sharifnia T, Antoun J, Verriere TG, Suarez G, Wattacheril J, Wilson KT, et al Hepatic TLR4 signaling in obese NAFLD. Am J Physiol Gastrointest Liver Physiol. 2015;309(4):G270–8. Epub 2015/06/27. 10.1152/ajpgi.00304.2014 ajpgi.00304.2014 [pii]. 26113297 PMC4537925

[40] He S, Liang Y, Shao F, Wang X. Toll-like receptors activate programmed necrosis in macrophages through a receptor-interacting kinase-3-mediated pathway. Proc Natl Acad Sci U S A. 2011;108(50):20054–9. Epub 2011/11/30. 10.1073/pnas.1116302108 1116302108 [pii]. 22123964 PMC3250173

[41] Levin S, Pevsner-Fischer M, Kagan S, Lifshitz H, Weinstock A, Gataulin D, et al Divergent levels of LBP and TGFbeta1 in murine MSCs lead to heterogenic response to TLR and proinflammatory cytokine activation. Stem Cell Rev. 2014;10(3):376–88. Epub 2014/03/26. 10.1007/s12015-014-9498-z . 24664302

[42] Mo IF, Yip KH, Chan WK, Law HK, Lau YL, Chan GC. Prolonged exposure to bacterial toxins downregulated expression of toll-like receptors in mesenchymal stromal cell-derived osteoprogenitors. BMC Cell Biol. 2008;9:52 Epub 2008/09/19. 10.1186/1471-2121-9-52 1471-2121-9-52 [pii]. 18799018 PMC2567970

[43] Tang J, Wu T, Xiong J, Su Y, Zhang C, Wang S, et al Porphyromonas gingivalis lipopolysaccharides regulate functions of bone marrow mesenchymal stem cells. Cell Prolif. 2015;48(2):239–48. Epub 2015/02/14. 10.1111/cpr.12173 . 25676907 PMC6496502

[44] Sodhi CP, Shi XH, Richardson WM, Grant ZS, Shapiro RA, Prindle T Jr., et al Toll-like receptor-4 inhibits enterocyte proliferation via impaired beta-catenin signaling in necrotizing enterocolitis. Gastroenterology. 2010;138(1):185–96. Epub 2009/09/30. 10.1053/j.gastro.2009.09.045 S0016-5085(09)01695-3 [pii]. 19786028 PMC2813409

