# Supplementary figures and images for: TLR4 Activation Promotes Bone Marrow MSC Proliferation and Osteogenic Differentiation via Wnt3a and Wnt5a Signaling
Source: PLoS One. 2016 Mar 1;11(3):e0149876. doi: 10.1371/journal.pone.0149876 (PMC4773221; doi:10.1371/journal.pone.0149876)

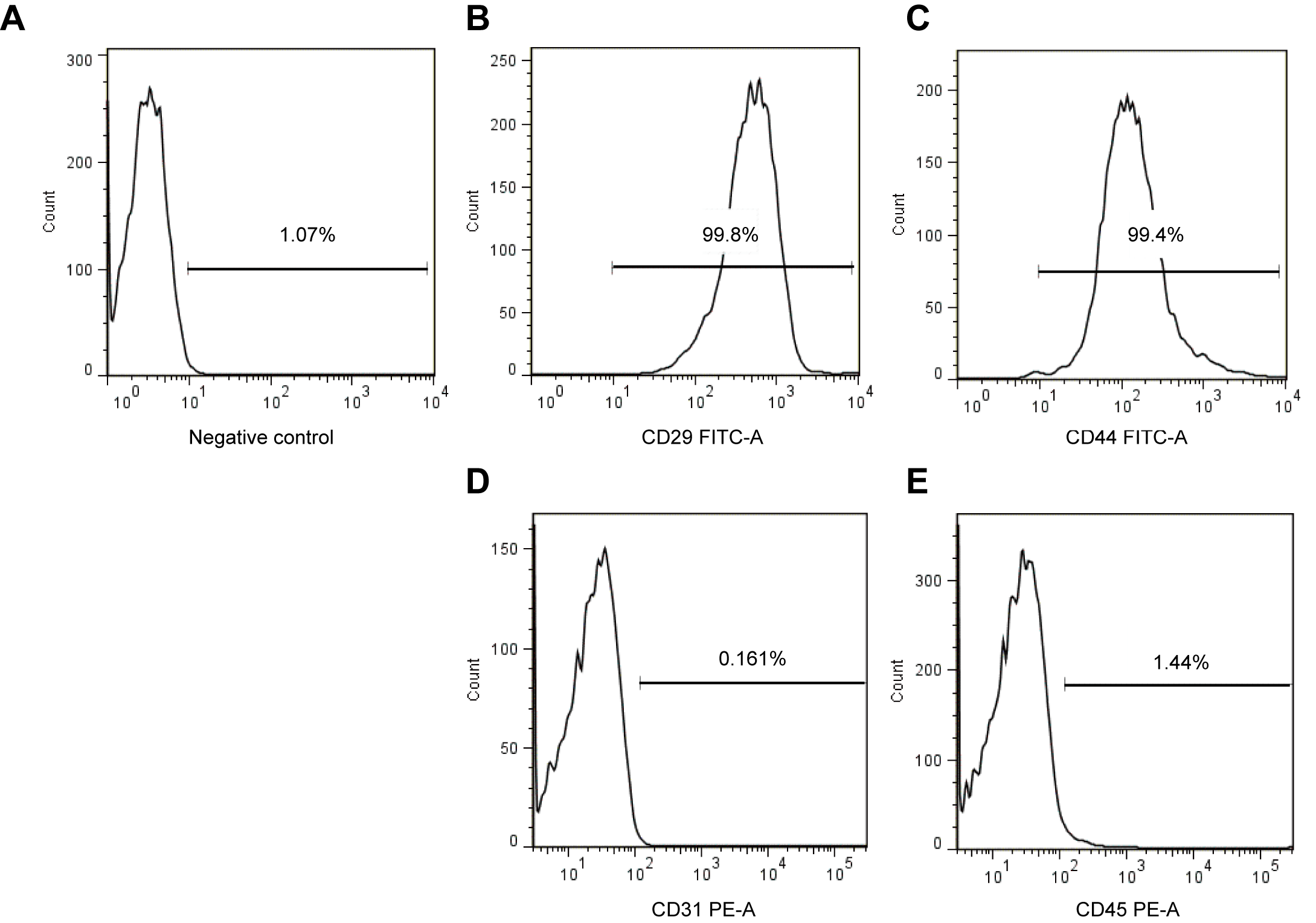

Supplement: S1 Fig — The flow cytometric analysis shows that the cultured cells are CD29 (B) and CD44 (C) positive. The cells are also CD31 (D) and CD45 (E) negative. (TIF) [file pone.0149876.s001.tif]

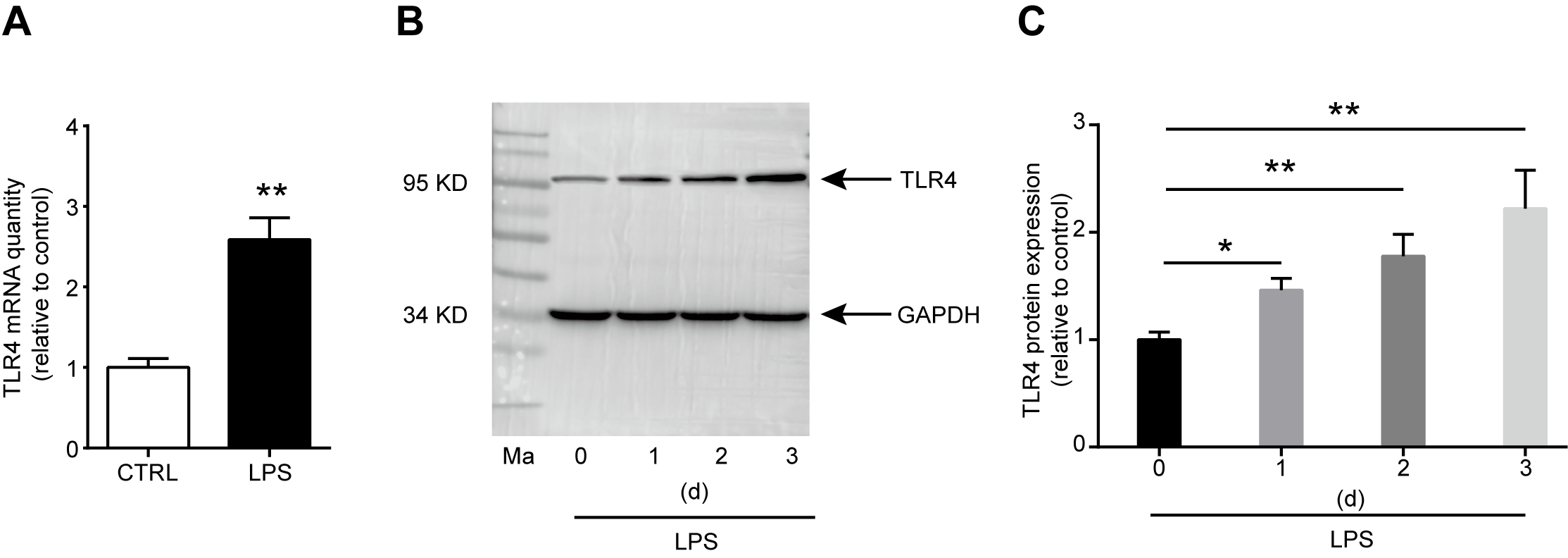

Supplement: S2 Fig — (A) The mRNA expression of TLR4 in MSCs was upregulated after 1000 ng/ml LPS treated for 3 days as detected by real-time PCR analysis. (B, C) The protein level of TLR4 is increased by LPS treatment as detected by western blotting analysis. Data are from three independent experiments and presented as mean ± SEM. *P<0.05, **P<0.01. (TIF) [file pone.0149876.s002.tif]

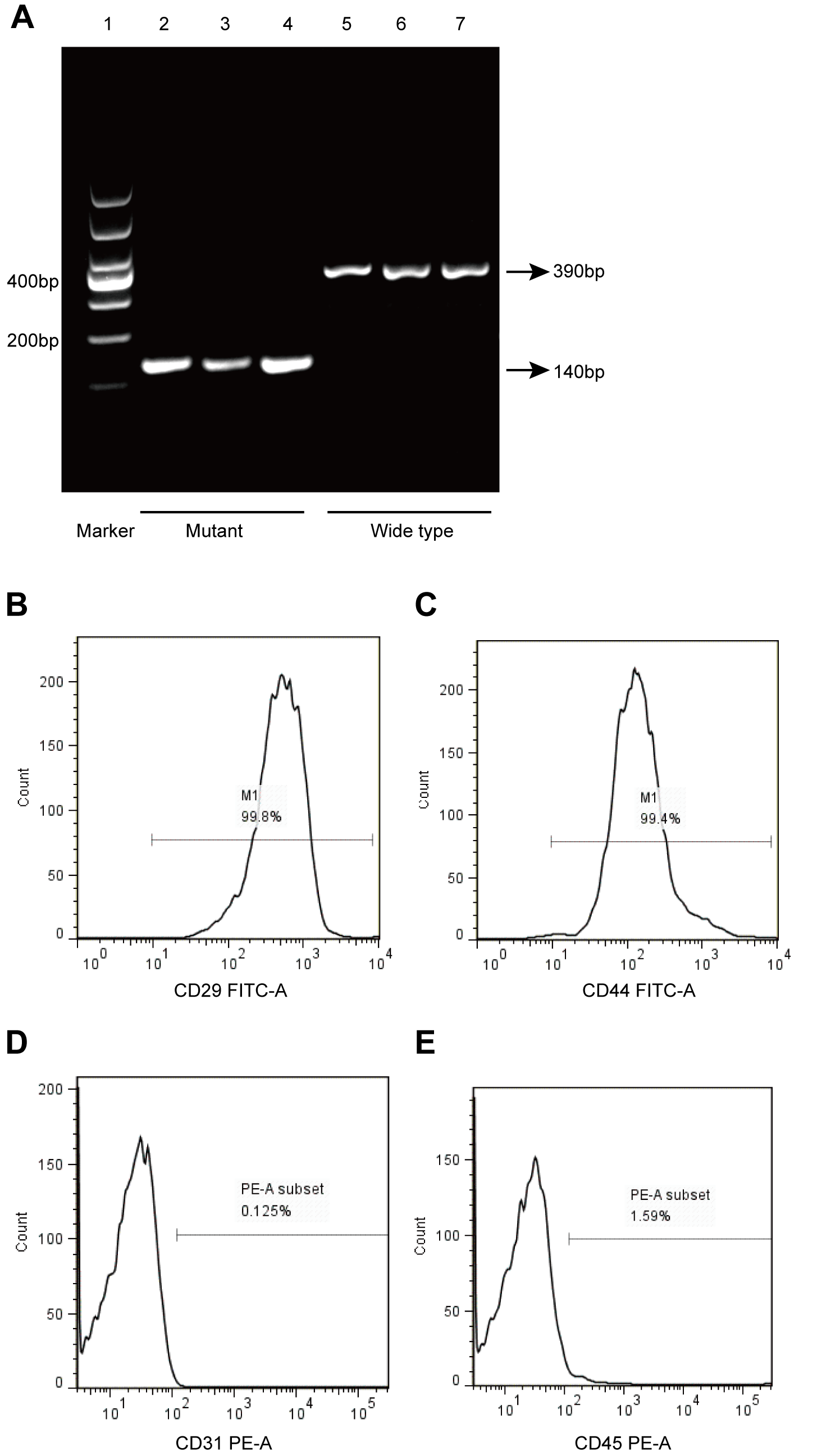

Supplement: S3 Fig — (A) PCR identification of TLR4 KO mice. Genomic DNAs were extracted from tails of the mouse and were analyzed by PCR using the following primers: wild-type TLR4 primer (5'ATATGCATGATCAACACCACAG 3' and 5' TTTCCATTGCTGCCCTATAG 3'), mutant TLR4 primer (5'GCAAGTTTCTATATGCATTCTC 3' and 5' CCTCCATTTCCAATAGGTAG 3'). The 140 bp band is the mutant TLR4 (lane 2 to 4), while the 390 bp band represented the wild-type genotype of TLR4 (lane 5 to 7). (B-E) Characteristic of bone marrow derived MSCs from TLR4 KO mice. The cultured cells are CD29+ (B), CD44+ (C), CD31 -(D), and CD45 -(E). (TIF) [file pone.0149876.s003.tif]

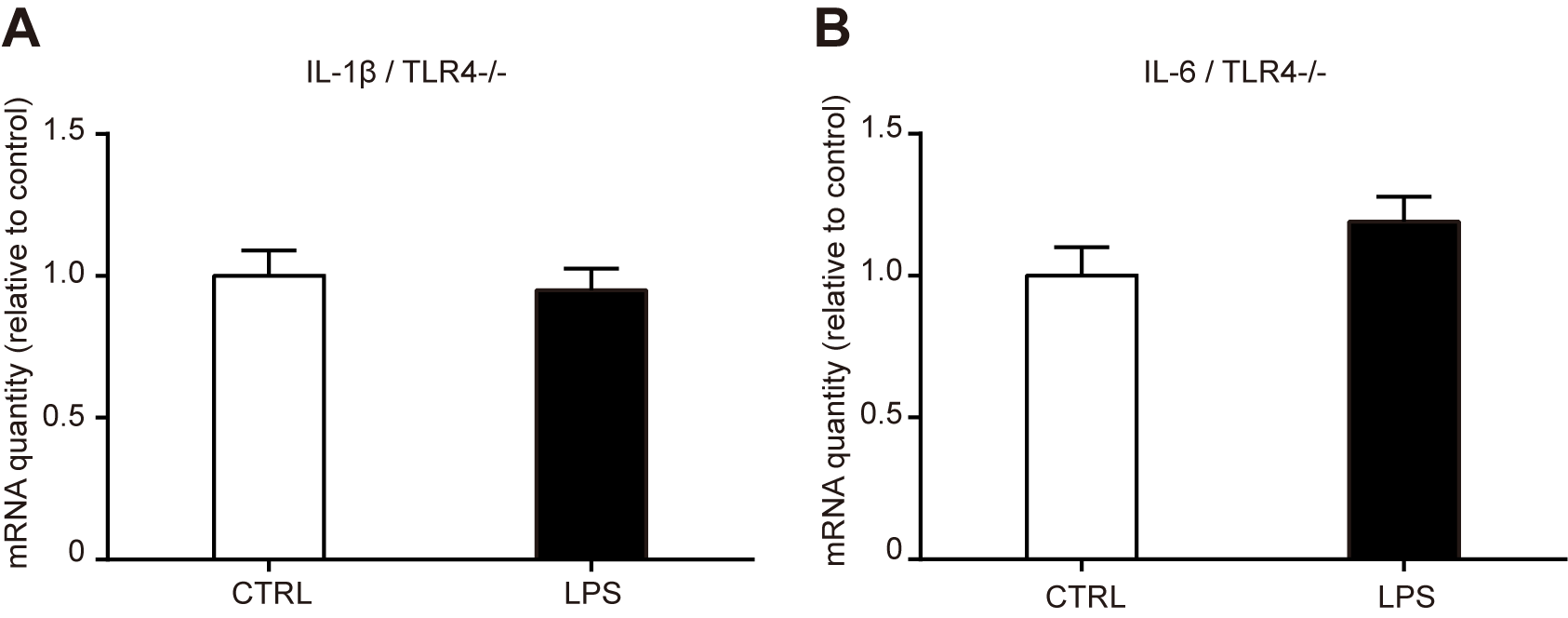

Supplement: S4 Fig — TLR4-/- MSCs were treated by 1000 ng/ml LPS for 3 days, the mRNA ecpression of IL-1β (A) and IL-6 (B) were detected by real-time PCR. No change was found in both of IL-1β and IL-6 mRNA expression. Data are from three independent experiments and presented as mean ± SEM. (TIF) [file pone.0149876.s004.tif]

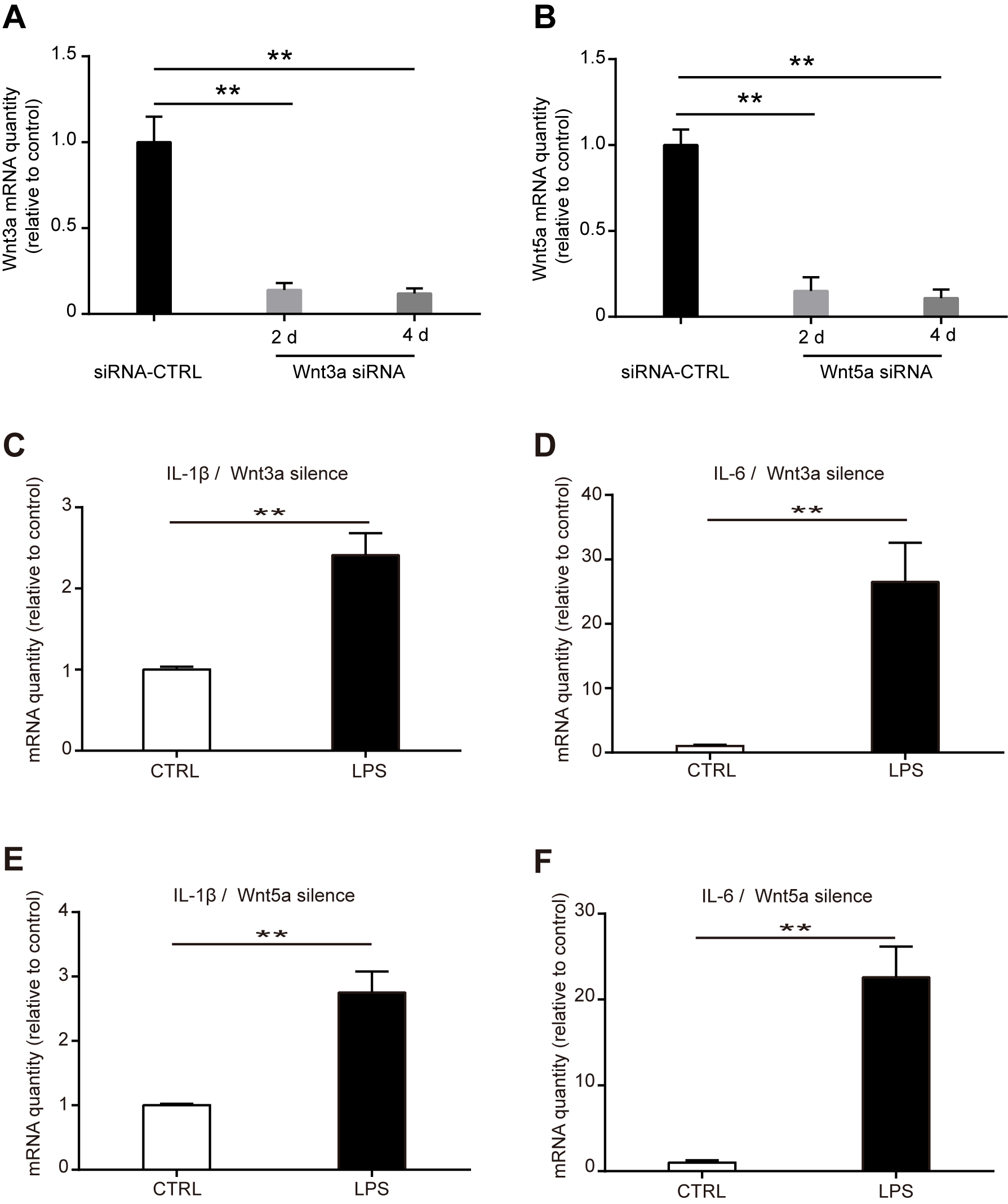

Supplement: S5 Fig — (A, B) Wnt3a and Wnt5a siRNA transfection effectively silence Wnt3a and Wnt5a expression. MSCs were transfected with Wnt3a and Wnt5a siRNA respectively. The mRNA expression of Wnt3a (A) and Wnt5a (B) was detected by real-time PCR 2 and 4 days after transfection. Data are from three independent experiments and presented as mean ± SEM. **P<0.01. (C-F) Effects of Wnt3a and Wnt5a silence on LPS-induced cytokine production in MSCs. Wild type MSCs were transfected with Wnt3a (C, D) or Wnt5a (E, F) siRNA respectively, the mRNA expression of IL-1β and IL-6 was then detected by real-time PCR. Data are from three independent experiments and presented as mean ± SEM. **P<0.01. (TIF) [file pone.0149876.s005.tif]

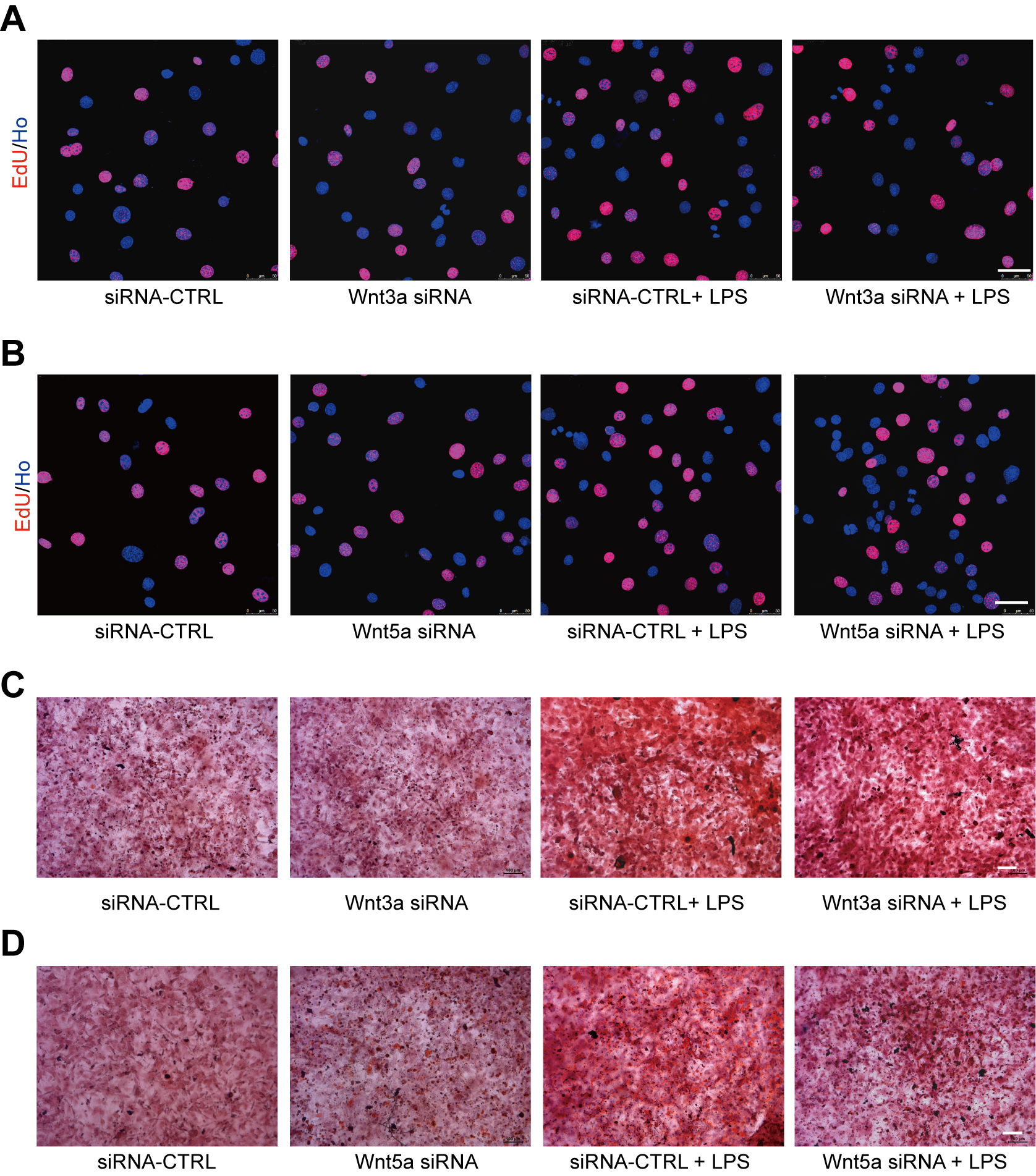

Supplement: S6 Fig — (A, B) Representative images of EdU incorporation as detected 6 days after LPS treatment in Wnt3a and Wnt5a silence cells. Scale bar: 50 μm. (C, D) Representative images of alizarin red staining as detected 15 days after LPS treatment. Scale bar: 100 μm. (TIF) [file pone.0149876.s006.tif]
